# The Hot Springs of Arkansas—Location—Character and Products of the Surrounding Country—Medicinal Qualities of the Waters, Etc.

**Published:** 1866-01

**Authors:** R. M. Lackey

**Affiliations:** Asst. Surg. and Brvt. Capt. U. S. Vols.; U. S. A. Gen’l Hospital, Little Rock, Ark.


*The Hot Springs of Arkansas—Location—Character and Products of the Surrounding Country—Medicinal Qualities of the Waters, etc.* By R. M. LACKEY, M. D., Asst. Surg. and Brvt. Capt. U. S. Vols.

Having determined upon a visit to the Hot Springs of Arkansas, the mild fair weather of October, and the dry roads, seemed especially favorable for a pleasant and profitable trip, and on the 13th we left this place for the Springs.

The Hot Springs are situated in Hot Springs County, sixty miles in a southwesterly direction from Little Rock. The country between Little Rock and the Springs is hilly and poor, the ground rocky and heavily timbered. Yellow pine, white, black and the Spanish oaks, and the black jack, constitute mainly the large growth, and paw paw, sassafras, dogwood, greenbrier, elder bushes, etc., the undergrowth.

The plantations on the road are scarce, and mostly on the bottom lands, along the larger streams. The small farms on the uplands are nearly all deserted, on account of the war in some cases, and in others, because too poor and unproductive to afford the inhabitants the means of subsistence. The people of this country partake of the character—physically, mentally and morally—that so invariably pertains to the poor whites of the South. The men are destitute of energy, industry and enterprise. The women, especially, bear the impress of that pathological state induced by the habitual and excessive use of tobacco in some form, bad food and malaria. They may be said to have neither form nor comeliness. Even in the mountainous districts of Arkansas, malarial diseases prevail very generally and in their most obstinate form. And this brings to mind a case of Arkansas surgery, of which I obtained some account. A lady came to a distinguished surgeon with a large abdominal tumor, which he diagnosed as ovarian. An operation for the removal of the tumor was decided upon, but what was the surprise of the surgeon to find, when coming down upon the tumor, that it was nothing more nor less than the woman's *spleen!* The enlarged condition and abnormal situation of the organ was easily accounted for when it was known that she had lived many years on the Washita River, where for people to have ague is the rule, and not to have it the exception.

In the immediate vicinity of Hot Springs the country is mountainous, the highest peaks reaching an altitude of five hundred feet above the valleys. On the southern flank of one of these mountains or ridges, are situated the Hot Springs. There are forty-two principal fountains from which the thermal waters issue; some of them are at the foot of the mountain, and the others scattered over its slope up to the heighth of one hundred feet above the creek, into which the waters flow from all of the springs. All of the forty-two springs are contained within the space of a few acres, and are so near together in some cases, that each hand may be placed in the water of a different spring at the same time; and what is remarkable in one instance, the waters of two of the springs that are so near together, differ greatly in temperature,—that of one being only one hundred degrees F., while the other, but three or four feet distant, is one hundred and thirty-four degrees F. The temperature of the waters at the fountain heads ranges from one hundred to one hundred and fifty degrees F. One would suppose, however, from dipping the hand into the hottest, that the water was almost at the boiling point, as the hand must be withdrawn instantly; and it surely is "scalding hot," for the quantity of hog hair seen near one of the springs, demonstrates that the water has been efficaciously used for scalding hogs; and the egg shells to be seen, are evidence that eggs may be cooked.

The steam arising from the water, and the constant escape of carbonic acid gas in bubbles, gives it a close resemblance to boiling water. The quantity of hot water issuing from these springs is immense; and so much water from so many different sources near together, splashing and gurgling down the steep mountain side, is grand and interesting. The creek formed by the water from all the springs is sufficient to afford a fine water-power; but no advantage has been taken of it as yet. The steam arising from the hot waters fills the valley early in the morning like a dense fog.

Hot Springs Ridge, on the side of which the springs are situated, seems to be mainly a mass of Novaculite rock. It is a beautiful white rock, resembling the finest varieties of marble, and is what is commonly known as "Arkansas Whetstone," or "Ouachita Oilstone." In company with Dr. G. W. Lawrence, a resident at the Springs, and to whom we are indebted for many courtesies and much valuable information, we visited a fine quarry, where this rock is taken out and hauled off to be made into whetstones. For surgical instruments and fine-edged tools, its grit is unsurpassed.

This Novaculite rock, which is so abundant throughout the mountainous portions of the State, belongs to the age of the millstone grit, and is composed of almost pure silica, as is shown by the following analysis taken from Owen's "Geological Survey:"

| | |
|---|---|
| Silica, in 100 parts, | 98.00 |
| Alumina, tinged with oxide of iron, | .80 |
| Soda, | .50 |
| Potash, | .60 |
| Traces of lime, magnesia and hydrofluoric acid and moisture, | .10 |
| | 100.00 |

That portion of the side of the mountain over which the waters flow is covered with calcareous tufa: near the foot of the mountain the accumulation of this tufa forms a high cliff, which, from the slowness of its formation by depositions from the water, must have been long ages in forming.

There is quite a thick growth of dwarfed trees covering the slope of the mountain, and vegetation grows within a very short distance of the hot water,—even in places upon these masses of calcareous tufa, bushes are growing wherever the hot water does not come in direct contact with their roots. The prickly pear, a species of cactus, especially grows quite luxuriantly on the tufaceous formations.

There are no igneous rocks visible within ten miles of the Springs, and the evidences of volcanic action are entirely wanting.

Some of the springs have been named in accordance with the medicinal substances they are reputed to contain in such quantities as to produce a decided effect upon the animal functions of those who use the water freely; and invalids who visit the place in search of health, drink immense quantities of the water with the firm belief that it contains the antidote for all their ills. For example, one is known as the "Arsenic Spring," another as the "Alum Spring;" but, according to any analyses that have yet been made, it does not appear that arsenic or alum are to be found in the waters of these springs, even in the minutest quantities. I give below the analysis of the so-called arsenic spring, taken from Owen's "Geological Survey."

In 1000 grammes of the water, the constituents were as follows:

| | Grammes. |
|---|---|
| Lime, | 0.059024 |
| Silicates, | 0.045600 |
| Sulphuric acid, | 0.019400 |
| Magnesia, | 0.007629 |
| Chlorine, | 0.002275 |
| Soda, | 0.004650 |
| Potash, | 0.001560 |

Not only do the ignorant people—the natives—attribute great medical virtues to the Hot Springs, but some of them believe, it would seem, that the waters of certain of the springs afford nutriment also; for a colored man, from a plantation near by, informed us, that if we would season the water of a certain spring that he pointed out, it would "taste jis like chicken broff." Sambo's story may have some shadow of truth, but his "chicken broff," I imagine, would be homeopathic in character, and could be appreciated only by the "little pill" doctors.

The waters of the Hot Springs are remarkable for their purity. They contain but very small quantities of mineral substances for spring water, and these consist mainly of silica and carbonate of lime. There is, however, small portions of iron in the water of some of the springs; and there are several chalybeate springs of cold water near by. One of these, "Fairchild's Chalybeate Spring," three miles distant, affords a large amount of water—enough to drive a small mill which had been erected to grind corn. There are sulphur springs also in the neighborhood.

Various theories have been advanced in regard to the cause of the high temperature of these waters. The opinion of Prof. Owen seems to be the most plausible. It is his belief that it is due to the "internal heat of the earth, that the waters are completely permeated by highly-heated vapors and gases which emanate from sources deeper-seated than the water itself."

It is evident, from the analyses of the waters, that their medical virtues are due to their high temperature mainly, if not altogether. All the mineral constituents most abundant are substances that have little or no action upon the animal functions. The large amount of carbonic acid gas contained in the water, it is thought, may produce an exhilirating effect on the system, and being so charged with this gas, invalids are enabled to drink large quantities of it without being nauseated, as is ordinarily the case from drinking large draughts of warm water. The water from the springs, from twenty-five to one hundred feet up the mountain, can be conducted in troughs to tanks above the bath-houses, and baths of any temperature desired can be had; while the water from the lower springs can be retained in tanks under the bath-rooms, so that the advantages of vapor baths may be obtained better than by any means ever invented or used by the "steam doctors." It is by a judicious system of bathing and steaming, causing a copious diaphoreses and arousing the absorbents, and the eliminative functions of the system, that the curative properties of these waters are to be obtained. The benefit to be derived must, however, be confined mainly to a certain class of disorders.

It is for rheumatic and syphilitic diseases that people mostly resort to these springs; some cases that seem to be purely of a neuralgic nature are, however, promptly relieved. There are probably more old syphilitic cases than any other form of disease that find their way there, and Dr. Lawrence, a very intelligent medical gentleman, who has treated an immense number of these cases, informed us that the judicious use of baths, in conjunction with other treatment indicated, proves very efficacious in old intractable cases of syphilis and rheumatism.

The beneficial results obtained, in many cases, are brought about mainly, I have no doubt, by administering to minds diseased. The scenery about the springs is wild and magnificent, and the springs themselves are certainly among the great natural wonders of this continent; and many who go there are impressed with the belief that anything so wonderful must possess great curative powers. The judicious use of electricity with these baths—constituting the electro-thermal bath—would, in my opinion, render them applicable to a much larger class of cases, and more efficacious.

Previous to the breaking out of the war, there were indifferent accommodations at the springs for seven or eight hundred persons, and we were informed that as many as one thousand visitors had been there at once. The buildings were, however, nearly all burned down about two years ago, and now the boarding and bathing accommodations are scarcely sufficient for one hundred persons. The title to the property, it seems, has not been settled, and there are several claimants. It is to be hoped that the United States will establish its claim to the property, and erect an hospital there for the treatment of the large number of rheumatic soldiers that are always to be found in our army. A recommendation of this kind was made to the

Surgeon General by Lt. Col. Jos. R. Smith, U. S. A., Med. Dir. Dep't of Ark., but was, I believe, disapproved at Washington. It is unfortunate as it is, for the improvement of the place is retarded, which is necessary to make it a beneficial place of resort for invalids, which I believe it is destined to be, despite the hilly, rocky, uninhabitable country to pass over in getting there.

A few miles from the Hot Springs, west, in Montgomery county, are the most extensive mines of rock crystals known on this continent. We procured some specimens of these masses of quartz crystals, that I imagine equal in brilliancy those of the Alps.

We also visited Magnet Cove, ten miles southeast from the springs, where large quantities of magnetic iron ore are found, together with an extensive variety of other minerals. The great quantities of iron pyrites (sulphuret of iron) found in this cove induced the so-called Confederate Government to erect an establishment there for the manufacture of sulphur with which to make gunpowder. We had the privilege of examining the first specimen that was made, which was a mass of tears just as it came from the retort. This was, I believe, the first of this article ever manufactured in the late Southern Confederacy. The quality of the sulphur was fair, and quite a quantity of it had been made and sent off before our army occupied that country.

Owen enumerates about twenty different varieties of minerals which are found in Magnet Cove, within an area of less than two miles. Much of the iron ore has polarity; titanic acid enters into its composition, and is also disseminated amongst the other minerals of the cove. In some parts of this cove the magnetic needle is strongly affected, and in the lower portions igneous rocks are found—the nearest ones to the Hot Springs.

Arkansas, though not now in a condition of physical, moral and political healthfulness, has vast mineral and other resources which the rapid advancement of the age will soon cause to be developed, and she may yet occupy an enviable position amongst her sister States of the Republic.

U. S. A. Gen'l Hospital, Little Rock, Ark., Nov. 10, 1865.

